# Post-mortem magnetic resonance imaging with computed tomography-guided biopsy for foetuses and infants: a prospective, multicentre, cross-sectional study

**DOI:** 10.1186/s12887-022-03519-4

**Published:** 2022-08-03

**Authors:** Christoph Martin Rüegger, Dominic Gascho, Peter Karl Bode, Elisabeth Bruder, Christian Haslinger, Steffen Ross, Kevin Schmid, Claudia Knöpfli, Lisa J. Hofer, Leonhard Held, Rosa Maria Martinez, Hans Ulrich Bucher, for the Virtopsy Study Group, Christoph M. Rüegger, Claudia Knöpfli, Hans Ulrich Bucher, Jean-Claude Fauchère, Brigitte M. Koller, Rosa M. Martinez, Steffen Ross, Christine Bartsch, Dominic Gascho, Peter K. Bode, Elisabeth Bruder, Christian Haslinger, Leonhard Schäffer, Kevin Schmid, Bernhard Frey, Lisa Hofer, Leonhard Held

**Affiliations:** 1 Newborn Research, Department of Neonatology, University Hospital Zurich, University of Zurich, Zurich, Switzerland; 2 Department of Forensic Medicine and Imaging, Institute of Forensic Medicine, University of Zurich, Zurich, Switzerland; 3 Department of Pathology, University Hospital and University of Zurich, Zurich, Switzerland; 4 Pathology, Institute of Medical Genetics and Pathology, University Hospital and University of Basel, Basel, Switzerland; 5 Department of Obstetrics, University Hospital and University of Zurich, Zurich, Switzerland; 6 Department of Intensive Care and Neonatology, University Children’s Hospital Zurich, Zurich, Switzerland; 7 Epidemiology, Biostatistics and Prevention Institute (EBPI), University of Zurich, Zurich, Switzerland

**Keywords:** Autopsy, Biopsy, Foetus, Infant, Magnetic resonance imaging, Minimally invasive, Post-mortem, Radiology, Virtual autopsy, Virtopsy

## Abstract

**Background:**

Post-mortem imaging has been suggested as an alternative to conventional autopsy in the prenatal and postnatal periods. Noninvasive autopsies do not provide tissue for histological examination, which may limit their clinical value, especially when infection-related morbidity and mortality are suspected.

**Methods:**

We performed a prospective, multicentre, cross-sectional study to compare the diagnostic performance of post-mortem magnetic resonance imaging with computed tomography-guided biopsy (Virtopsy®) with that of conventional autopsy in foetuses and infants. Cases referred for conventional autopsy were eligible for enrolment. After post-mortem imaging using a computed tomography scanner and a magnetic resonance imaging unit, computed tomography-guided tissue sampling was performed. Virtopsy results were compared with conventional autopsy in determining the likely final cause of death and major pathologies. The primary outcome was the proportion of cases for which the same cause of death was determined by both methods. Secondary outcomes included the proportion of false positive and false negative major pathological lesions detected by virtopsy and the proportion of computed tomography-guided biopsies that were adequate for histological examination.

**Results:**

Overall, 101 cases (84 fetuses, 17 infants) were included. Virtopsy and autopsy identified the same cause of death in 91 cases (90.1%, 95% CI 82.7 to 94.5). The sensitivity and specificity of virtopsy for determining the cause of death were 96.6% (95% CI 90.6 to 98.8) and 41.7% (95% CI 19.3 to 68.0), respectively. In 32 cases (31.7%, 95% CI 23.4 to 41.3), major pathological findings remained undetected by virtopsy, and in 45 cases (44.6%, 95% CI 35.2 to 54.3), abnormalities were diagnosed by virtopsy but not confirmed by autopsy. Computed tomography-guided tissue sampling was adequate for pathological comments in 506 of 956 biopsies (52.7%) and added important diagnostic value in five of 30 cases (16.1%) with an unclear cause of death before autopsy compared with postmortem imaging alone. In 19 of 20 infective deaths (95%), biopsies revealed infection-related tissue changes. Infection was confirmed by placental examination in all fetal cases.

**Conclusions:**

Virtopsy demonstrated a high concordance with conventional autopsy for the detection of cause of death but was less accurate for the evaluation of major pathologies. Computed tomography-guided biopsy had limited additional diagnostic value.

**Trial registration:**

ClinicalTrials.gov (NCT01888380).

**Supplementary Information:**

The online version contains supplementary material available at 10.1186/s12887-022-03519-4.

## Background

In view of declining autopsy rates around the world, post-mortem imaging, including computed tomography (CT) and magnetic resonance imaging (MRI), has been reported as an acceptable alternative to conventional autopsy in foetuses and infants [1–5]. Even though CT and MRI can provide accurate macroscopic information about internal structures, histological and microbiological tissue evaluation is not possible with these methods. This is a major disadvantage of every imaging-based, noninvasive post-mortem examination, especially when infection-related morbidity and mortality are suspected [6].

A number of minimally invasive procedures have been tested for their contributions to the diagnostic accuracy of post-mortem imaging [7, 8]. Percutaneous needle biopsies have been proposed with and without image guidance, but studies in the perinatal period revealed that the majority of samples taken from smaller foetal organs were inadequate for histological examination [9–11]. To facilitate the identification and examination of organs while minimizing incisions, a minimally invasive autopsy with whole-body MRI and laparoscopically assisted tissue sampling was assessed in a small feasibility study of perinatal cases [12]. Although additional diagnostic information was provided in the majority of cases compared with MRI alone, further evaluation of minimally invasive autopsy in a large number of cases is required. Percutaneous needle biopsies under CT guidance have been suggested to be a more reliable method for targeted sampling of tissue probes in adults [13–15]. However, its feasibility in the paediatric population has not yet been evaluated.

In the current study, we tested the hypothesis that, for foetuses and infants, post-mortem MRI with CT-guided needle biopsy (Virtopsy®) would provide similar diagnostic information to that of conventional autopsy for determining the cause of death (COD) and major pathological abnormalities.

## Methods

### Study design and population

This interdisciplinary validation study was prospectively registered at Clinicaltrials.gov (NCT01888380) and approved by the Ethics Committee Zurich (KEK-ZH-Nr: 2014–0286). Prospective written informed parental consent was obtained. The study protocol has been described previously [16].

Between January 2014 and June 2018, foetuses (≤ 24 weeks’ or > 24 weeks’ gestation) and infants (aged < 12 months) from four academic institutions (Departments of Obstetrics and Neonatology of the University Hospital Zurich, Department of Intensive Care and Neonatology of the University Children’s Hospital Zurich, and Institute of Forensic Medicine and Imaging of the University of Zurich) referred for a conventional autopsy were eligible for enrolment. The exclusion criteria were a gestational age (GA) at birth below 16 weeks, a birth weight below 150 g, and a postnatal age above 12 months [16].

### Procedures

Virtopsy involved whole-body imaging with a 128-slice CT (Somatom Definition; Siemens Medical Solutions, Forchheim, Germany) and a 3 Tesla MRI scanner (Philips Achieva; Philips Medical Systems, Best, The Netherlands) followed by CT-guided biopsies. All MRI and CT images were evaluated and reported by a forensic radiologist (S.R. – 10 years of foetal post-mortem imaging experience) masked to the autopsy findings. In line with previous publications, small foetal ventricular bleeds, and small amounts of pleural, pericardial, or peritoneal fluid were classified as intrapartum or post-mortem imaging changes with no clinical significance [3, 17]. In foetal cases, hypoxic-ischaemic brain lesions were regarded as the final common pathway of normal post-mortem changes unless a specific preceding hypoxic event was identified.

CT-guided biopsies were performed using a robotic system (B-Rob II, iSYS Medizintechnik GmbH and Austrian Centre for Medical Innovation and Technology ACMIT, Austria) [18]. Tissue was extracted with an automatic biopsy pistol (Bard Magnum, Bard Biopsy Systems, Tempe, AZ, USA) including a coaxial introducer needle (13-gauge × 10.3 cm) and a biopsy core needle (14-gauge × 16 cm). Irrespective of suspected abnormality on CT and MRI, biopsies of the same organs (adrenal gland, heart, kidney, lung, liver, spleen, brain, pancreas, thymus) were attempted in all cases by a trained member of the virtopsy team (R.M.M.—13 years of experience in paediatric forensic pathology). Specimens were fixed in formalin and examined by an independent pathologist (E. B.—25 years of experience in paediatric pathology) who was blinded to the results of conventional autopsy.

Conventional autopsies and examinations of the placenta were performed by an experienced perinatal and paediatric pathologist (P.K.B.—10 years of experience in paediatric pathology) as soon as possible after virtopsy [19]. The pathologist was aware of the clinical history and all relevant antemortem information but masked to the virtopsy results.

### Data collection and outcomes

For each case, COD and the most important pathological abnormality were reported at three sequential time points using a previously published classification system [20]: after clinical evaluation, after virtopsy, and after conventional autopsy. Independent access to the database was provided for each timepoint, blinded to the results of autopsy (Table 1).

**Table 1:** Available information for cause of death evaluation at each sequential time point

**CLINICAL EVALUATION** [clinician]	**VIRTOPSY** [forensic radiologist, forensic pathologist, paediatric pathologist]		**AUTOPSY** [paediatric pathologist]
	**Imaging**	**Histopathology**	
Patient history	** + **Post-mortem MRI	** + **CT-guided biopsies	Patient history
Clinical examination	** + **Post-mortem CT	** + **Placental examination	Fetoplacental examination
Laboratory testing			Block histology
Genetic testing^a^			Laboratory testing
Ultrasound			Genetic testing^a^
Foetal MRI^a^			Conventional X-ray

At each time point, independent access to the database was provided (e.g., the clinician was blinded to the results of virtopsy and autopsy, the virtopsy team was blinded to the results of autopsy)

^a^In selected cases

COD and major pathologies determined at each sequential timepoint were defined as concordant or discordant in two successive reviews by two experts (CMR and HUB) not involved in any of the cases. Disagreements were resolved by consensus.

The primary outcome, namely, concordance between virtopsy and autopsy, was defined as the proportion of cases for which virtopsy identified the same COD as conventional autopsy (gold standard). Post hoc analyses of the primary outcome were performed in two specified subgroups: in cases with an unclear COD after clinical evaluation and in cases with infective deaths. Secondary outcomes included sensitivity, specificity, and predictive values of clinical evaluation and virtopsy referenced to the gold standard, the proportion of false positive (overcalled) and false negative (undetected) major pathological findings per organ system detected by virtopsy, and the proportion of CT-guided biopsies that were adequate for histological examination.

### Analysis

Concordance was calculated with a 95% Wilson confidence interval (CI). A Newcombe CI for paired data was used to assess the difference between two proportions [21]. The mean and standard deviation (SD) are reported for continuous outcomes and the median and interquartile range (IQR) for non-parametrical outcomes. A convenience sample of 100 cases was needed to determine the primary outcome within 8% with 95% confidence if concordance was 80%. The width of the CI decreased to ± 6% if concordance was 90%.

## Results

**Fig. 1:**
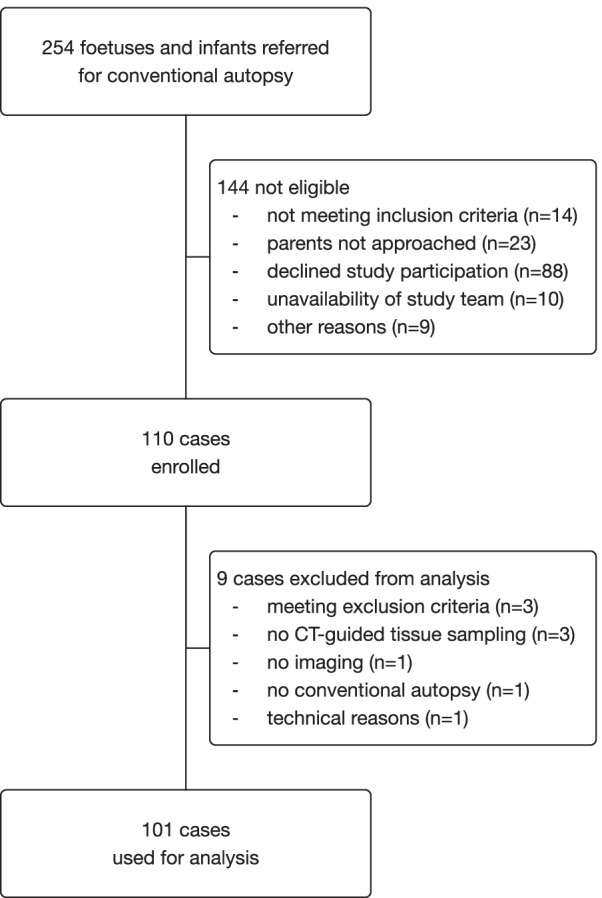
Number of cases who were screened, enrolled, and included in the primary analysis

Data from 101 cases were included in the study (Fig. 1). Eighty-four (83%) were foetuses, and 17 (17%) were infants (Table 2).

**Table 2:** Clinical characteristics of the study population

	**Foetuses**		**Infants**	**Total**
	≤ 24 weeks GA	> 24 weeks GA		
n (%)	58 (57)	26 (26)	17 (17)	101 (100)
Female, n (%)	26 (45)	17 (65)	6 (35)	49 (49)
Median gestational age (IQR), wks	21.5 (20.0 – 23.0)	30.5 (26.0 – 33.0)	34 (29.3—37.5)	23.4 (21.6—28.6)
Median age at death (IQR), days	0 (0—0)	0 (0—0)	9 (1—49)	0 (0—0)
Median weight at death (IQR), g	370 (273—520)	1285 (728—2450)	2140 (1433—3185)	520 (320—988)
Median length at death (IQR), cm	18 (16.0—20.6)	28 (22.5—33.0)	37 (28.0—38.0)	21 (18.0—28.0)

*IQR* Interquartile range, *GA* Gestational age

In the foetal group, termination of pregnancy was performed in 44 (52%) cases (induction of labour in 38 cases, feticide by ultrasound-guided intracardiac KCL injection in 6 cases). In 24 (29%) foetal cases, intrauterine death was unexplained after clinical evaluation. The mean (SD) post-mortem intervals between death/birth and virtopsy and between death/birth and autopsy were 49 (29) and 78 (32) hours, respectively.

### Cause of death

COD was identified in 71 (70%), 93 (92%), and 95 (94%) cases based on clinical, virtopsy, and autopsy findings. Virtopsy and autopsy identified the same COD in 91 cases (90.1%, 95% CI 82.7 to 94.5), with a higher concordance for foetuses than for infants (difference 16.4%, 95% CI 0.5 to 40.4). In 63 cases (62%), the virtopsy team suggested that a full conventional autopsy was unnecessary. In this subgroup, concordance with autopsy was 93.7% (95% CI 84.8 to 97.5).

In 30 cases (30%), COD was unclear after clinical evaluation (26 foetuses, 4 infants). None of the 30 cases resolved with post-mortem imaging alone, but virtopsy was concordant with autopsy in 23 of the 30 cases (76.7%, 95% CI 59.1 to 88.2). In the seven discordant cases, three infective deaths were not detected by virtopsy, and in three cases, placental abnormality was not judged as the primary COD by the virtopsy team. In 17 of the 26 unclear foetal cases (65%), placental abnormalities were present. In 20 cases (20%), deaths were infection related (Table 3). In this subgroup, agreement between virtopsy and autopsy was 85.0% (95% CI 64.0 to 94.8).

**Table 3:** Significant biopsy and block histology findings in infective deaths

**ID**	**Age group** (Cause of death)	**CT-guided biopsy**	**Block histology**	**Placenta**
2	**Infant** (Sepsis, Kawasaki disease)	Heart: myocarditis/Liver: ICIs	Pancarditis/ICIs	N/A
6	**Infant** (Sepsis)	Lung: pneumonia	Necrotizing pneumonia	Distal villous hypoplasia
7	**Infant** (Sepsis)	Lung: ICIs/Liver: ICIs	Haemorrhagic oedema/ICIs	N/A
9	**Infant** (Sepsis)	Lung: alveolar haemorrhages Colon: ischemic colitis	Alveolar haemorrhages NEC	N/A
13	**Infant** (Sepsis)	Lung: ICIs and alveolar haemorrhages	Congestion	N/A
14	**Foetus** (IAI)	Lung: ICIs	ICIs	Chorioamnionitis
24	**Foetus** (IAI)	Lung: ICIs	ICIs	Chorioamnionitis
30	**Foetus** (IAI)	Lung: ICIs	ICIs	Chorioamnionitis
31	**Foetus** (IAI)	Lung: ICIs	ICIs	Chorioamnionitis
38	**Infant** (Encephalitis)	Brain: not adequate for comment	Encephalitis	No placental examination
42	**Foetus** (CMV infection)	Heart: owl’s-eye cells Liver: cytomegalic cells	Intranuclear and Intracytoplasmic inclusions	Placental hydrops with CMV inclusions
66	**Infant** (NEC, Sepsis)	Colon: NEC with purulent peritonitis	NEC with purulent peritonitis	Chorioamnionitis
72	**Foetus** (IAI)	Lung: ICIs	ICIs	Chorioamnionitis
75	**Foetus** (IAI)	Lung: ICIs	Normal	Chorioamnionitis
78	**Foetus** (IAI)	Lung: ICIs	ICIs	Chorioamnionitis
84	**Foetus** (IAI)	Lung: ICIs	Normal	Chorioamnionitis
93	**Foetus** (IAI)	Lung: ICIs	ICIs	Chorioamnionitis
101	**Foetus** (IAI)	Lung: ICIs	Normal	Chorioamnionitis
102	**Foetus** (IAI)	Lung: ICIs	ICIs	Chorioamnionitis
103	**Foetus** (IAI)	Lung: ICIs	ICIs	Chorioamnionitis

*ID* Study identification number, *ICIs* Inflammatory cell infiltrates, *NEC* Necrotizing enterocolitis, *IAI* Intraamniotic infection, *CMV* Cytomegalovirus, *N/A* Not applicable

Concordance rates and diagnostic accuracy for each method, overall and by age group, are shown in Table 4.

**Table 4:** Cause of death: Diagnostic accuracy and concordance compared with conventional autopsy

	**TP** n	**FP** n	**TN** n	**FN** n	**Concordance** % (95% CI)	**Sensitivity** % (95% CI)	**Specificity** % (95% CI)	**PPV** % (95% CI)	**NPV** % (95% CI)
**All cases (*n* = 101)**									
Virtopsy	86	7	5	3	90.1 (82.7 to 94.5)	96.6 (90.6 to 98.8)	41.7 (19.3 to 68.0)	92.5 (85.3 to 96.3)	62.5 (30.6 to 86.3)
Clinical evaluation	59	12	6	24	64.4 (54.6 to 73.0)	71.1 (60.6 to 79.7)	33.3 (16.3 to 56.3)	83.1 (72.7 to 90.1)	20.0 (9.5 to 37.3)
**Foetuses ≤ 24 weeks (*n* = 58)**									
Virtopsy	50	2	4	2	93.1 (83.6 to 97.3)	96.2 (87.0 to 98.9)	66.7 (30.0 to 90.3)	96.2 (87.0 to 98.9)	66.7 (30.0 to 90.3)
Clinical evaluation	36	7	4	11	69.0 (56.2 to 79.4)	76.6 (62.8 to 86.4)	36.4 (15.2 to 64.6)	83.7 (70.0 to 91.9)	26.7 (10.9 to 52.0)
**Foetuses > 24 weeks (*n* = 26)**									
Virtopsy	23	1	1	1	92.3 (75.9 to 97.9)	95.8 (79.8 to 99.3)	50.0 (9.5 to 90.5)	95.8 (79.8 to 99.3)	50.0 (9.5 to 90.5)
Clinical evaluation	12	3	1	10	50.0 (32.1 to 67.9)	54.5 (34.7 to 73.1)	25.0 (4.6 to 69.9)	80.0 (54.8 to 93.0)	9.1 (1.6 to 37.7)
**Infants (*n* = 17)**									
Virtopsy	13	4	0	0	76.5 (52.7 to 90.4)	100.0 (77.2 to 100.0)	0.0 (0.0 to 49.0)	76.5 (52.7 to 90.4)	NaN
Clinical evaluation	11	2	1	3	70.6 (46.9 to 86.7)	78.6 (52.4 to 92.4)	33.3 (6.1 to 79.2)	84.6 (57.8 to 95.7)	25.0 (4.6 to 69.9)
**Cases with unclear cause of death after clinical evaluation (*n* = 30)**									
Virtopsy	18	4	5	3	76.7 (59.1 to 88.2)	85.7 (65.4 to 95.0)	55.6 (26.7 to 81.1)	81.8 (61.5 to 92.7)	62.5 (30.6 to 86.3)
**Cases with infective deaths (*n* = 20)**									
Virtopsy	17	3	0	0	85.0 (64.0 to 94.8)	100.0 (81.6 to 100.0)	0.0 (0.0 to 53.1)	85.0 (64.0 to 94.8)	NaN
Clinical evaluation	7	7	6	0	35.0 (18.1 to 56.7)	53.8 (29.1 to 76.8)	0.0 (0.0 to 35.4)	50.0 (26.8 to 73.2)	0.0 (0.0 to 39.0)

*TP* True positive, *FP* False positive, *TN* True negative, *FN* False negative, *PPV* Positive predictive value (= TP/TP + FP), *NPV* Negative predictive value (= TN/TN + FN), *NaN* Not a number

### Major pathological findings

Virtopsy identified 150 major pathological findings. In 45 cases (44.6%, 95% CI 35.2 to 54.3), 58 findings (39%) were not confirmed by autopsy (overcalled). The proportion of cases with at least one overcalled pathology decreased from infants (58.8%, 95% CI 36.0 to 78.4) to foetuses > 24 weeks GA (50.0%, 95% CI 32.1 to 67.9) and foetuses ≤ 24 weeks GA (37.9%, 95% CI 26.6 to 50.8). Overcalled pathologies were most often related to the heart (16%—mainly structural abnormalities), brain (16%—minor intraventricular bleeds, agenesis of corpus callosum, hypoxic-ischemic injuries), lungs (12%—pulmonary hypoplasia, pneumothorax), and gastrointestinal system (7%).

Conventional autopsy detected 138 major pathological findings. In 32 cases (31.7%, 95% CI 23.4 to 41.3), 36 findings (19%) remained undetected by virtopsy. The proportion of cases with at least one undetected pathology decreased from infants (47.1%, 95% CI 26.2 to 69.0) to foetuses > 24 weeks GA (42.3%, 95% CI 25.5 to 61.1) and foetuses ≤ 24 weeks GA (22.4%, 95% CI 13.6 to 34.7). Undetected pathologies were most frequent in the lung (15%—mainly pulmonary hypoplasia and meconium aspiration), gastrointestinal system (8%—intestinal atresia and necrotizing enterocolitis), and heart (7%—structural abnormalities and cardiomyopathy).

A complete list of overcalled and undetected abnormalities identified at virtopsy is shown in additional file 1. Concordance for major pathological abnormalities across different organ systems is provided in Table 5.

**Table 5:** Concordance across organ systems for major pathological findings

	**TP** n	**TN** n	**x**	**N**	**Concordance** % (95% CI)
**Lung**					
Virtopsy	24	50	74	101	73.3 (63.9 to 80.9)
Clinical evaluation	14	57	71	101	70.3 (60.8 to 78.3)
**Heart**					
Virtopsy	14	64	78	101	77.2 (68.1 to 84.3)
Clinical evaluation	9	75	84	101	83.2 (74.7 to 89.2)
**Brain**					
Virtopsy	20	47	67	86^a^	77.9 (68.1 to 85.4)
Clinical evaluation	10	57	67	86^a^	77.9 (68.1 to 85.4)
**Gastrointestinal system**					
Virtopsy	6	80	86	101	85.1 (76.9 to 90.8)
Clinical evaluation	2	86	88	101	87.1 (79.2 to 92.3)
**Urogenital system**					
Virtopsy	16	80	96	101	95.0 (88.9 to 97.9)
Clinical evaluation	11	82	93	101	92.1 (85.1 to 95.9)
**Musculoskeletal system**					
Virtopsy	12	84	96	101	95.0 (88.9 to 97.9)
Clinical evaluation	8	86	94	101	93.1 (86.4 to 96.6)

^a^removal of the brain with examination after fixation was not performed in 15 cases

*TP* True positive, *TN* True negative, *x* TP + TN

### CT-guided biopsies

Of 956 organ biopsies attempted from 9 target organs, 506 (53%) were adequate for histological examination. In 77 of those biopsies (15%), no subsequent histological examination of the same organ was performed during autopsy, leaving 429 biopsies (85%) for further comparison. In 308 of 429 CT-guided biopsies (72%), the findings agreed with the block histology (Table 6).

**Table 6:** CT-guided biopsy; Success rate and comparison with block histology

	**CT-guided biopsy**			**Block histology**
	**Attempted** n	**Successful** n (%)	**Biopsies for which block histology was available** n (%)	**Findings confirmed** n (%)
Overall	956	506 (53)	429 (85)	308 (72)
Brain	74	49 (66)	19 (39)	5 (26)
Lung^a^	221	147 (67)	133 (90)	79 (59)
Thymus	78	18 (23)	16 (89)	12 (75)
Heart	118	84 (71)	75 (89)	66 (88)
Liver	130	112 (86)	102 (91)	85 (83)
Kidney^a^	120	52 (43)	48 (92)	35 (73)
Adrenal gland^a^	77	20 (26)	17 (85)	11 (65)
Pancreas	61	10 (16)	8 (80)	8 (100)
Spleen	77	14 (18)	11 (79)	7 (64)

^a^Both sides

Among the 30 cases with an unexplained COD after clinical evaluation, CT-guided biopsies provided significant histopathological abnormalities in five cases (17%—three foetuses with perinatal infection, two infants with sepsis). In all three foetal cases, infection was confirmed by placental examination.

Among the 20 infective deaths, CT-guided biopsies provided evidence of inflammatory cell infiltrates in the lung, heart, liver, and gut in 19 cases (95%—13 foetal cases with signs of perinatal infection, 6 infants with sepsis and/or necrotizing enterocolitis – Table 3). In an infant with viral encephalitis, brain biopsy was not adequate for histological examination. CT-guided biopsies of the lung provided infection-related tissue changes in 16 cases (80%). In the foetal age group, the information obtained from the placenta confirmed infection in all cases.

## Discussion

Virtopsy demonstrated a high concordance with conventional autopsy for the detection of the COD among foetuses and infants. In cases for which the virtopsy team predicted that a full autopsy was unnecessary, concordance further increased, illustrating the potential significance of virtopsy as a suitable alternative to autopsy in selected cases when invasive post-mortem examination is not possible or not desired. While CT-guided biopsies confirmed infection-related tissue changes in the majority of infective deaths, they did not provide useful additional diagnostic information that was not apparent from other examinations, such as placental pathology. Together with a low biopsy success rate and a moderate informative value of corresponding histopathological samples, the overall contribution of CT-guided biopsy was low, resulting in the high sensitivity and low specificity of our virtual autopsy approach.

Previous studies in foetuses and infants concentrated on the performance of post-mortem MRI alone, offering a high diagnostic accuracy similar to that reported in our study [1]. The largest prospective comparison evaluating a minimally invasive autopsy procedure (MRI, examination of the placenta, post-mortem blood sampling) with conventional autopsy in foetuses and children is the Magnetic Resonance Imaging Autopsy (MaRIAS) study [3]. While the MaRIAS study demonstrated a high concordance with conventional autopsy for the detection of COD or major pathological lesions in foetuses and children, it was less accurate in older children due to the increase in infective anomalies across this age range. Our study aimed to compensate for this limitation with the addition of CT-guided biopsies for histological diagnosis, but we found that virtopsy did not further increase the diagnostic yield compared with previously published results evaluating a minimally invasive autopsy or post-mortem imaging alone. We speculate that this may be due to the following reasons.

**Fig. 2:**
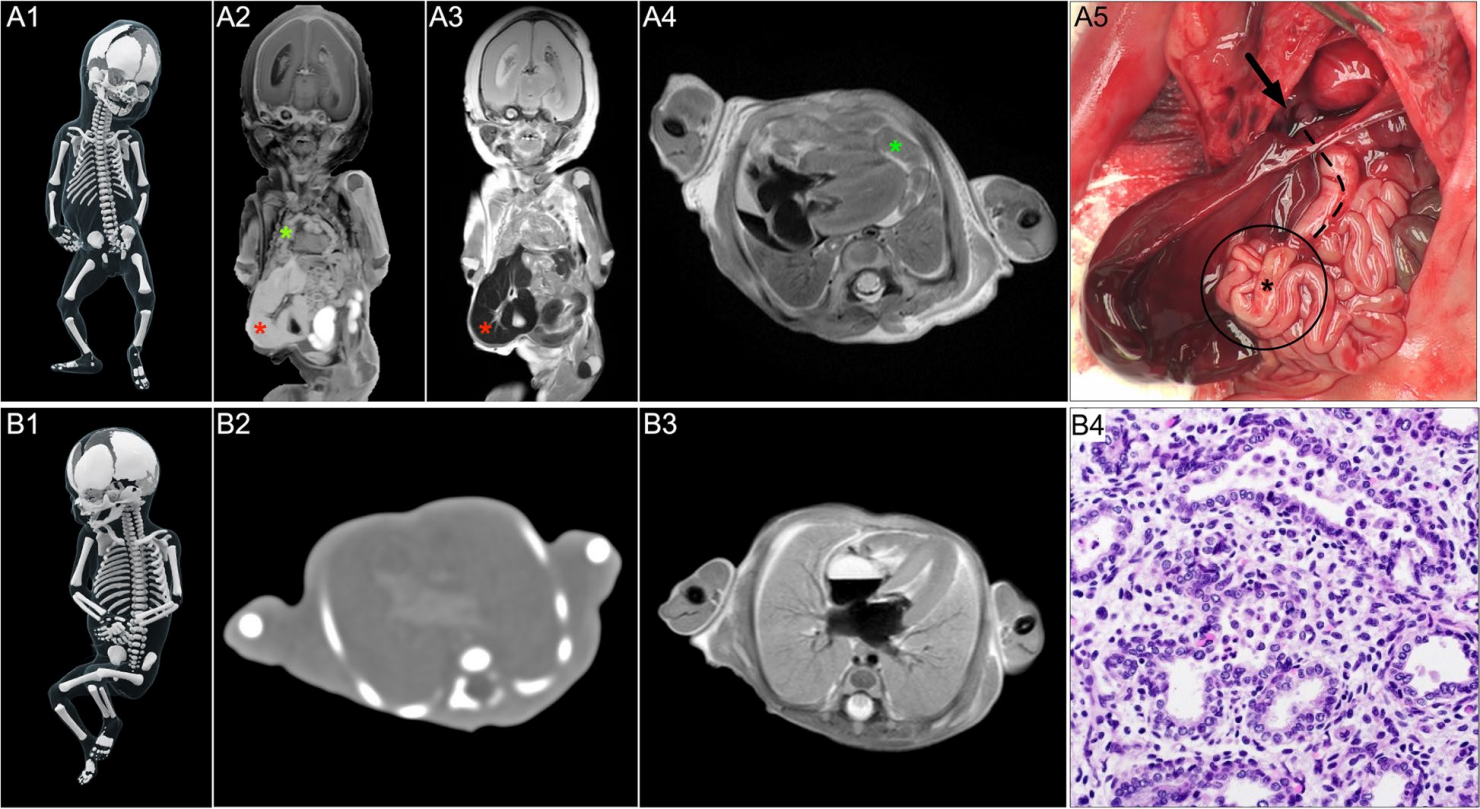
Virtopsy and autopsy findings of two exemplary foetal cases with and without structural abnormalities. **A** Post-mortem computed tomography (CT) and magnetic resonance imaging (MRI) of a 22 weeks foetus with pentalogy of Cantrell. **A1** CT—cinematic rendered reconstruction of skeletal appearance. **A2** and **A3** MRI—T1 inversion recovery and T2 turbo spin echo coronal views: exomphalos with herniation of liver (red asterisk). Herniation of small bowel loops through an absent diaphragmatic portion of the pericardium into the pericardium (green asterisk). **A4** MRI—T2 turbo spin echo transversal view: herniation of small bowel loops into the pericardium (green asterisk). **A5** thoraco-abdominal autopsy—reduction of bowel loops (black asterisk) through the anterior pericardial defect (black arrow) into the abdomen. **B** Post-mortem CT and MRI of a 20 weeks foetus without structural abnormalities born after premature rupture of membranes and uterine contractions. **B1** CT—cinematic rendered reconstruction of skeletal appearance. **B2** CT—soft kernel transversal view: poor soft tissue contrast in the foetal population, making assessment of the thoracic and abdominal organs almost impossible. **B3** MRI—T2 turbo spin echo transversal view: better contrast and clearer differentiation of the foetal intra-thoracic organs. **B4** CT—guided biopsy: haematoxylin and eosin staining with evidence of inflammatory cell infiltrates in the lung

First, the majority of subjects in our study were foetuses in whom structural abnormalities were prevalent. Structural abnormalities can usually be assessed with imaging alone; thus, tissue for histopathological examination is not required. In foetuses without structural abnormalities, perinatal infections were common. Although most infective cases showed histological abnormalities on CT-guided biopsies, the correct diagnosis could have been made without microscopic examination of foetal organs based on other noninvasive investigations, such as placental examination [22, 23]. Therefore, the diagnostic role of routine tissue sampling for the investigation of foetal death is limited and should be performed only in selected cases when foetal and perinatal infection is suspected, and placental examination is not possible. In our study, foetal lung tissue with evidence of inflammatory cell infiltrates was most sensitive for perinatal infection and should thus be the biopsy of choice in such a scenario (Fig. 2).

Second, our biopsy sampling rate was low, with only half of biopsies confirmed histologically to be of the respective target organ. Sampling failure was most frequent in organs with reduced soft tissue contrast on CT, such as the spleen, pancreas, and adrenal glands. This is a well-known disadvantage of CT, particularly in the foetal and neonatal population due to reduced abdominal and subcutaneous fat, making assessment of the small thoracic and abdominal cavity organs almost impossible. Post-mortem micro-CT has been shown to be a viable and useful tool in the foetal age group, offering excellent image resolution near the histological level. However, implementation of this technique may be hampered by the limited availability of micro-CT in routine clinical practice [2]. Previous biopsy studies in foetuses and infants assessed the sampling performance of blind percutaneous [9, 24], ultrasound-guided percutaneous [25], and laparoscopic [12] biopsies as part of a minimally invasive autopsy. Unsurprisingly, rates of adequate histological sampling were highest under direct visualization during laparoscopic autopsy for most organs [12]. Percutaneous biopsies with and without image guidance provided lower biopsy success, similar to the one reported in our study. This is in contrast to the adult population, where CT-guided biopsies were successfully obtained as part of a minimally invasive autopsy procedure [13].

Finally, a significant proportion of histological abnormalities detected with CT-guided biopsies could not be confirmed by block histology. Since agreement rates for histological abnormalities improve with increasing biopsy sample size, we may have missed the most representative parts with the relatively small amount of tissue that we collected [26].

Overcalled pathologies were most frequent in the heart, brain, and lung. In the heart, overcalled diagnoses were related to structural abnormalities, most likely due to the complexity of the cardiac anatomy and the limited resolution of CT and MRI, especially in the lower foetal age group [27]. In the brain, hypoxic-ischemic lesions were present in almost all cases. Their differentiation into antemortem ischemic brain injuries and post-mortem cerebral changes is a known weakness of MRI. Nevertheless, MRI is accepted as an accurate investigational technique for identifying significant neuropathology in the perinatal and paediatric periods, even in cases where autolysis prevents conventional neuropathological examination [28]. Therefore, we speculate that some cerebral lesions overcalled by virtopsy may in fact represent undetected findings at autopsy. Overcalls in the lung were related mainly to pulmonary hypoplasia and pneumothorax. While pulmonary hypoplasia is best defined by antenatal ultrasound, the ratio of lung weight to body weight, or radial alveolar counting during post-mortem examination, MRI has not yet become an accepted method for predicting pulmonary hypoplasia in foetuses at high risk [29–31]. Since post-mortem imaging appears more useful than conventional autopsy for the identification of air leaks, we hypothesize that some of the smaller pneumothoraxes overcalled at virtopsy may also represent undetected findings at autopsy [13].

Undetected pathologies were more frequent in infants than in foetuses. While this is in line with previous reports [3], we noted no excess in infection-related abnormalities among undetected pathologies. This finding contrasts with the results of the MaRIAS study, which reported a low accuracy for minimally invasive autopsy in children because of missed infections of the lungs or heart [3]. Our study demonstrated a high diagnostic yield for CT-guided biopsies in infective cases, resulting in a relatively low number of infection-related abnormalities undetected at virtopsy.

This is the first study to evaluate the feasibility and diagnostic value of CT-guided biopsies in foetuses and infants. The prospective study design with multicentre participation and differing patient populations increases the representativeness of the results. Moreover, all findings were reported in a blinded fashion to obtain an objective measure of diagnostic accuracy. Our study has some limitations. First, we used a robotic system with an image-guided surgical navigation system for CT-guided tissue sampling. Although unique in the perinatal and postnatal setting and possibly less operator dependent than other biopsy methods, the results of such a highly technical tissue sampling method may be difficult to replicate in less specialized settings. Since a single forensic paediatric pathologist performed all CT-guided biopsies, inter-operator variability could not be evaluated. Second, all cases were reported by experienced forensic radiologists and pathologists with special education in this field. This may not represent what could be offered in other institutions. Third, only cases with consent for conventional autopsy were included, which resulted in a population that may be unrepresentative for cases that may benefit from virtopsy in the future. Fourth, infants were poorly represented in our study, which may underestimate the overall informative value of biopsies in our cohort of mostly noninfective cases. Finally, we did not perform a comparative evaluation of the amount of work and costs for virtopsy and conventional autopsy.

## Conclusions

Virtopsy provided similar diagnostic information to that of conventional autopsy for determining COD in foetuses and infants but was less accurate for the identification of major pathological abnormalities. CT-guided biopsies improved the diagnostic value of CT and MRI in specific situations but, overall, provided little diagnostic information that was not apparent from other examinations. Our results add to the growing evidence that post-mortem imaging with or without additional minimally invasive procedures may be offered as an alternative to conventional autopsy in selected situations.

## Supplementary Information

**Additional file 1.** Overcalled and undetected abnormalities identified at virtopsy.

**Additional file 2.** Virtopsy Study Group.

## Data Availability

The datasets used and/or analysed during the current study are available from the corresponding author on reasonable request.

## Abbreviations

CI: Confidence interval
COD: Cause of death
CT: Computed tomography
GA: Gestational age
IQR: Interquartile range
MRI: Magnetic resonance imaging
SD: Standard deviation
