# Supplementary material for: Post-mortem magnetic resonance imaging with computed tomography-guided biopsy for foetuses and infants: a prospective, multicentre, cross-sectional study
Source: BMC Pediatr. 2022 Aug 3;22:464. doi: 10.1186/s12887-022-03519-4 (PMC9347089; doi:10.1186/s12887-022-03519-4)
Supplement: Supplementary file 1 — Additional file 1. Overcalled and undetected abnormalities identified at virtopsy. [file 12887_2022_3519_MOESM1_ESM.pdf]

Additional file 1: overcalled and undetected abnormalities identified at virtopsy

Lung			
**ID**	**Age group**	**False positive (overcalled)**	**False negative (undetected)**
6	Infant	Pulmonary interstitial emphysema	-
7	Infant	Aspiration	-
8	Foetus >24 weeks	-	Lung hypoplasia
9	Infant	Pneumothorax	-
10	Foetus ≤24 weeks	-	Lung hypoplasia
12	Foetus ≤24 weeks	-	Cystic hygroma
15	Foetus >24 weeks	-	Meconium aspiration
16	Foetus >24 weeks	-	Meconium aspiration
21	Foetus ≤24 weeks	Other	-
23	Foetus ≤24 weeks	Lung hypoplasia	-
29	Foetus ≤24 weeks	-	Lung hypoplasia
30	Foetus ≤24 weeks	-	Aspiration
31	Foetus ≤24 weeks	-	Aspiration
32	Infant	Pneumothorax	-
38	Infant	Aspiration	-
39	Foetus >24 weeks	-	Meconium aspiration
43	Foetus >24 weeks	Lung hypoplasia	-
47	Foetus ≤24 weeks	Lung hypoplasia	-
49	Foetus ≤24 weeks	-	Lung hypoplasia
55	Foetus ≤24 weeks	Lung hypoplasia	-
56	Foetus >24 weeks	-	Meconium aspiration
59	Foetus ≤24 weeks	-	Lung hypoplasia
60	Foetus >24 weeks	-	Lung hypoplasia
66	Infant	Lung lesion	-
69	Foetus >24 weeks	-	Meconium aspiration
79	Foetus >24 weeks	-	Meconium aspiration
94	Foetus ≤24 weeks	Lung hypoplasia	-

Heart			
ID	Age group	False positive (overcalled)	False negative (undetected)
8	Foetus >24 weeks	Other cardiac abnormality	-
11	Foetus ≤24 weeks	-	Common arterial trunk
12	Foetus ≤24 weeks	Hypoplastic left heart syndrome	-
17	Infant	-	Cardiomyopathy
18	Infant	Cardiomegaly	-
19	Infant	Cardiomegaly	-
36	Infant	-	Myocardial infarction
38	Infant	-	Coarctation
44	Foetus ≤24 weeks	Ventricular septal defect	-
48	Foetus >24 weeks	Cardiomegaly	-
58	Foetus >24 weeks	Atrioventricular septal defect	-
60	Foetus >24 weeks	Atrial septal defect	-
65	Infant	-	Dilated cardiomyopathy
68	Foetus ≤24 weeks	Complex congenital heart disease	-
69	Foetus >24 weeks	Biventricular hypertrophy	-
74	Foetus ≤24 weeks	Partial anomalous venous drainage	-
77	Foetus >24 weeks	Ventricular septal defect	-
82	Foetus ≤24 weeks	-	Ventricular septal defect
86	Foetus ≤24 weeks	Coarctation	-
91	Foetus ≤24 weeks	-	Ventricular septal defect
93	Foetus ≤24 weeks	Cardiomegaly	-
106	Foetus >24 weeks	Ventricular septal defect	-
107	Foetus >24 weeks	Atrial septal defect	-

Gastrointestinal system			
**ID**	**Age group**	**False positive (overcalled)**	**False negative (undetected)**
2	Infant	Splenomegaly	-
3	Foetus >24 weeks	-	Intestinal atresia
9	Infant	-	Necrotising enterocolitis
13	Infant	-	Necrotising enterocolitis
27	Foetus >24 weeks	-	Gastroenteritis
30	Foetus ≤24 weeks	-	Meckel's Diverticulum
47	Foetus ≤24 weeks	-	Malrotation
52	Foetus >24 weeks	-	Intestinal atresia
53	Foetus ≤24 weeks	Malrotation	-
58	Foetus >24 weeks	Other	-
60	Foetus >24 weeks	Dilated gut	-
67	Foetus ≤24 weeks	-	Intestinal atresia
73	Foetus ≤24 weeks	No spleen	-
89	Foetus ≤24 weeks	Exomphalos	-
102	Foetus ≤24 weeks	Malrotation	-

Brain			
**ID**	**Age group**	**False positive (overcalled)**	**False negative (undetected)**
2	Infant	-	Infection
3	Foetus >24 weeks	Callosal agenesis	-
8	Foetus >24 weeks	Neural tube defect	-
12	Foetus ≤ 24 weeks	Callosal agenesis	-
13	Infant	Asphyxia	-
14	Foetus ≤24 weeks	Intracranial bleed	-
21	Foetus ≤24 weeks	Callosal agenesis	-
38	Infant	-	Infection
44	Foetus ≤24 weeks	Callosal agenesis	-
48	Foetus >24 weeks	Asphyxia	-
49	Foetus ≤24 weeks	Intracranial bleed	-
61	Foetus ≤24 weeks	-	Ischemic brain injury
77	Foetus >24 weeks	Vermis hypoplasia	-
82	Foetus ≤24 weeks	Intracranial bleed	-
84	Foetus ≤24 weeks	Intracranial bleed	-
96	Foetus ≤24 weeks	Callosal agenesis	-
101	Foetus ≤24 weeks	Subdural bleed	-
108	Foetus >24 weeks	Vascular malformation	-
110	Foetus >24 weeks	Intracranial bleed	-

Urogenital system			
ID	Age group	False positive (overcalled)	False negative (undetected)
38	Infant	-	Renal developmental abnormality
42	Foetus ≤24 weeks	Renal agenesis	-
56	Foetus >24 weeks	Renal developmental abnormality	-
69	Foetus >24 weeks	-	Uterus agenesis
74	Foetus ≤24 weeks	Renal dysplasia	-

Musculoskeletal system			
ID	Age group	False positive (overcalled)	False negative (undetected)
7	Infant	-	Fracture long bones
21	Foetus ≤24 weeks	Skeletal dysplasia	-
32	Infant	Fracture skull	-
44	Foetus ≤24 weeks	Fracture long bones	-
69	Foetus >24 weeks	Other	-

Placenta			
ID	Age group	False positive (overcalled)	False negative (undetected)
22	Foetus ≤24 weeks	-	Infarction/insufficiency
33	Foetus ≤24 weeks	-	Placental abruption
43	Foetus >24 weeks	-	Placental abruption
84	Foetus ≤24 weeks	Infarction/insufficiency	-
101	Foetus ≤24 weeks	Chorioamnionitis/funisitis	-
102	Foetus ≤24 weeks	Chorioamnionitis/funisitis	-
103	Foetus ≤24 weeks	Chorioamnionitis/funisitis	-
110	Foetus >24 weeks	-	Other
