# Supplementary material for: Post-mortem magnetic resonance imaging with computed tomography-guided biopsy for foetuses and infants: a prospective, multicentre, cross-sectional study
Source: BMC Pediatr. 2022 Aug 3;22:464. doi: 10.1186/s12887-022-03519-4 (PMC9347089; doi:10.1186/s12887-022-03519-4)
Supplement: Supplementary file 2 — Additional file 2. Virtopsy Study Group. [file 12887_2022_3519_MOESM2_ESM.pdf]

Additional file 2: Virtopsy Study Group

First and middle names, last names	Affiliation
Christoph Martin, Rüegger	Newborn Research, Department of Neonatology, University Hospital and University of Zurich, Switzerland
Hans Ulrich, Bucher	
Claudia, Knöpfli	
Brigitte Maria, Koller	
Jean-Claude, Fauchère	
Rosa Maria, Martinez	Institute of Forensic Medicine, University of Zurich, Switzerland
Steffen, Ross	
Christine, Bartsch	
Dominic, Gascho	
Peter Karl, Bode	Department of Pathology, University Hospital and University of Zurich, Switzerland
Elisabeth, Bruder	Pathology, Institute of Medical Genetics and Pathology, University Hospital and University of Basel, Switzerland
Christian, Haslinger	Department of Obstetrics, University Hospital and University of Zurich, Switzerland
Leonhard, Schäffer	Department of Obstetrics, Cantonal Hospital Baden, Switzerland
Kevin, Schmid	Department of Intensive Care and Neonatology, University Children’s Hospital and University of Zurich, Switzerland
Bernhard, Frey	
Lisa, Hofer	Epidemiology, Biostatistics and Prevention Institute, University of Zurich, Switzerland
Leonhard, Held	
